# Touch, temperature, and relief: a multilevel integrative model of hedonic regulation in massage and thermal therapies

**DOI:** 10.3389/fphys.2026.1888366

**Published:** 2026-07-16

**Authors:** Vladislav Ruchkin, Roman A. Koposov

**Affiliations:** 1 Child and Adolescent Psychiatry Unit, Department of Medical Sciences, Uppsala University, Uppsala, Sweden; 2 Child Study Center, Yale School of Medicine, New Haven, CT, United States; 3 Sala Forensic Psychiatric Clinic, Sala, Sweden; 4 Regional Centre for Child and Youth Mental Health and Child Welfare, Faculty of Health Sciences, UiT The Arctic University of Norway, Tromsø, Norway; 5 Sechenov First Moscow State Medical University, Moscow, Russia

**Keywords:** affective touch, autonomic regulation, biopsychosocial model, interoception, massage therapy, neurochemical modulation, pain modulation, placebo and expectancy

## Abstract

Touch, massage, and thermal practices are widely used to promote relaxation, pain relief, and bodily restoration. Yet the mechanisms that make these practices feel pleasurable or restorative are usually explained in separate literatures, including somatosensory neuroscience, pain modulation, autonomic physiology, psychoneuroendocrinology, placebo research, and social psychology. This article proposes the Multilevel Integrative Model of Hedonic Regulation (MIHR) as a conceptual framework for linking these domains. The model synthesizes established findings on tactile and thermal signaling, descending modulation, reward and stress systems, and contextual meaning, while adding a testable account of how pleasure and relief arise when sensory input, bodily state, regulation, and context become aligned for the recipient. The same pressure, stroking velocity, warmth, or cold may feel soothing, neutral, intrusive, or aversive depending on expectation, perceived control, interpersonal trust, cultural familiarity, prior experience, and current bodily state. Relief is central to this account. In massage, sauna, bathing, and contrast exposure, positive affect often comes not only from pleasant stimulation itself, but from the easing of tension, pain, cold, heat, or bodily unease. The MIHR is presented as a testable framework rather than a validated mechanism. Future studies should manipulate sensory and contextual variables together, distinguish anticipation, stimulation, offset, and recovery phases, and combine subjective reports with autonomic, endocrine, and neurobiological measures. The model also separates immediate hedonic pleasure from broader eudaimonic wellbeing, which may emerge when repeated practices support recovery, agency, social connection, and a more secure relation to the body.

## Introduction

Touch is one of the basic ways in which humans encounter the world, regulate arousal, and maintain social bonds across the lifespan (Jakubiak and Feeney, 2017; Cascio et al., 2019). Practices built around touch and temperature, including massage, bathing, sauna, balneotherapy, and contrast exposure, have long been used to cultivate comfort, recovery, and wellbeing (An et al., 2019; Geri et al., 2019; Castelli et al., 2022). Their appeal is familiar at the level of experience but less settled at the level of mechanism. Why should pressure, warmth, stroking, immersion, or carefully bounded discomfort come to feel not only noticeable, but soothing, relieving, or even pleasurable?

Part of the difficulty is that the relevant evidence has grown in neighboring but only partly connected fields. Affective-touch research has emphasized C-tactile (CT) afferents and the social qualities of gentle stroking (McGlone et al., 2014; Ackerley, 2022). Pain science has focused on spinal gating, descending control, and endogenous analgesia. Clinical literature often reports benefits of touch-based therapies while leaving mechanisms comparatively open (Field, 2014; Packheiser et al., 2024). This fragmentation keeps alive a basic dispute: does pleasant touch originate mainly in specialized afferent pathways, especially CT fibers, or is it better understood as an experience assembled from discriminative touch, interoception, bodily state, expectation, appraisal, and cultural framing acting together (McGlone et al., 2014; Ellingsen et al., 2015; Marshall and McGlone, 2020; Case et al., 2023).

The present review follows the second, assembly-based view, while not diminishing the importance of peripheral specialization. Its contribution is to treat massage, bathing, sauna, and thermal contrast not as separate interventions, but as related practices that may produce pleasure and relief through overlapping sensory, spinal, interoceptive, autonomic, neurochemical, and social processes. In other words, a massage stroke, a hot bath, a sauna session, or the offset of cold exposure is not just a stimulus. It is a bodily event whose hedonic value is worked out across several levels at once. Pleasant touch is unlikely to be the signature of a single afferent class, cortical site, or transmitter. It is a lived regulatory experience built at the crossing of skin and peripheral channels, spinal and brainstem circuits, cortical appraisal, neurochemical tone, autonomic shifts, and the meanings people bring to the encounter. Under safe and predictable conditions, even stimulation that borders on discomfort, such as deep pressure or thermal contrast, can engage inhibitory systems and become associated with relief and, at times, pleasure (Navratilova and Porreca, 2014). This coupling is common in practice, but it is not universal and likely varies with history, context, and state.

The Multilevel Integrative Model of Hedonic Regulation (MIHR) proposed in the present review makes three main claims. First, tactile and thermal pleasure should be studied as an interaction among sensory input, bodily state, regulatory mechanisms, and contextual meaning. Second, relief should be treated as a distinct hedonic process rather than as a secondary afterthought of pain reduction. Third, individual differences in safety, control, prior experience, and cultural familiarity are central to whether touch or temperature becomes pleasant, neutral, or aversive.

The review develops this position with attention to massage, sauna, bathing, and contrast therapy. We move from peripheral encoding to spinal and subcortical modulation, then to cortical evaluation, neurochemical systems, autonomic regulation, and psychosocial context. The order is anatomical, but the argument is not linear: the experience of pleasure is shaped by feedback among these levels (Pessoa, 2017). Variability is not treated here as noise. The same pressure or heat can soothe one person and unsettle another, depending on prediction, trust, perceived control, bodily state, and prior experience.

An evolutionary perspective gives this variability a broader frame. Caregiving touch and grooming regulate stress, support affiliation, and signal safety in primates (Harlow, 1958; Dunbar, 2010; Griesser et al., 2025). Human developmental evidence, including skin-to-skin contact and kangaroo mother care, similarly shows that touch can stabilize arousal, support parent-infant co-regulation, and combine physiological regulation with relational meaning (Field, 2010; Bieleninik et al., 2021; Dreisoerner et al., 2021; Kostilainen et al., 2021). What remains less clear is how far professional touch and ritualized thermal practices recruit the same systems as intimate or affiliative touch. Some overlap seems likely, but professional touch may also substitute ritual order, explicit consent, skilled attunement, and learned associations of safety for the intimacy of close bonds.

Because the article brings together terms from somatosensory neuroscience, pain science, autonomic physiology, and affective science, key technical terms are defined in **Box 1**. The main text then uses these terms more compactly, returning to their functional role rather than redefining them at each appearance. We also distinguish three experiential outcomes at the outset. Hedonic pleasure refers to immediate positive sensory-affective experience; relief refers to positive affect that follows the reduction or offset of discomfort; and eudaimonic wellbeing refers to broader gains in meaning, agency, recovery, connectedness, or bodily confidence.

Box 1 **Key technical terms used in the article**

**Table:** 

Term	Intuitive meaning in this article
Aβ fibers	Fast, myelinated touch fibers that provide detailed information about location, pressure, texture, vibration, and movement. They are mainly discriminative rather than intrinsically affective.
C-tactile (CT) afferents	Slow, unmyelinated fibers found primarily in hairy skin that respond preferentially to gentle, slow, skin-temperature stroking and are strongly linked to affective touch.
TRPV1 and TRPM8	Temperature-sensitive ion channels involved respectively in heat/noxious heat and cool/cold signaling.
Dorsal horn	The first major spinal relay where touch, temperature, and nociceptive signals are filtered and combined.
Gate control/spinal gating	A mechanism by which non-painful touch can reduce transmission of pain-related input in the spinal cord, especially through inhibitory interneurons.
PAG-RVM network	The periaqueductal gray and rostral ventromedial medulla, a descending pain-control system linking brain evaluation to spinal nociceptive transmission.
Bounded discomfort	A stimulus that may be mildly unpleasant while it occurs, such as cold or firm pressure, but is voluntary, predictable, time-limited, and therefore able to become relieving after offset.
Cellular stress responses	Slower adaptive processes, such as heat-shock protein activity, that help cells manage thermal and metabolic challenge and may contribute to longer-term recovery rather than immediate pleasure.
Latent-profile approach	A statistical strategy for identifying subgroups of responders from patterns across several variables, rather than assuming that all participants respond in the same way.
Interoception	Sensing and interpreting the internal state of the body, including temperature, arousal, visceral sensations, and bodily comfort or discomfort.
Heart rate variability (HRV)	Variation in the time interval between heartbeats. It is often used as an index of autonomic regulation but is influenced by respiration, posture, movement, and recording conditions.
HPA axis	The hypothalamic-pituitary-adrenal stress system, commonly indexed through cortisol and related measures of stress physiology.
Predictive processing	The idea that the brain interprets incoming bodily signals in relation to prior expectations and updates those expectations when the signals do not fit.
Hedonic pleasure, relief, and eudaimonic wellbeing	Hedonic pleasure refers to immediate positive feeling; relief to positive affect after reduction or offset of discomfort; and eudaimonic wellbeing to longer-term meaning, agency, recovery, and connectedness.

A related clarification is needed for multilevel concepts such as safety, anxiety, boredom, anger, and depression. These terms do not designate a single fixed level of the model. In one design, perceived safety may be a contextual moderator that changes whether touch is interpreted as care or intrusion; in another, momentary felt safety may mediate the effect of predictable touch on autonomic settling; in a third, safety may be an outcome state, indexed by subjective calm, reduced vigilance, or willingness to repeat the practice. The same applies to negative affective states: anxiety or anger may be baseline moderators, intermediate appraisal states, or outcomes after stimulation. The empirical task is therefore to specify the role of each construct in a given hypothesis rather than to assume that it always belongs to one box of the model.

The MIHR should therefore be read as a set of linked hypotheses rather than as a claim that all mechanisms matter equally in every case. Much of the model synthesizes established findings; some of its cross-level links are more speculative and should be tested directly. Sensory stimulation is usually the experimental input; pleasure, relief, calm, pain reduction, and willingness to repeat the practice are key outcomes; and the intervening systems described below may explain, shape, or qualify the pathway between them. If contextual and person-level factors do not improve prediction beyond narrower single-mechanism accounts under well-controlled conditions, the model should be revised rather than simply expanded.

This is a conceptual narrative review rather than a systematic review. We did not attempt to estimate pooled treatment effects. Instead, we selected literatures that are central to the proposed framework: affective touch, massage therapy, thermal and balneological practices, pain modulation, interoception, autonomic physiology, reward and stress systems, placebo research, and cultural forms of bathing and sauna use. The goal is to organize plausible mechanisms and generate testable propositions, not to claim that all proposed links are already established.

## Peripheral mechanisms

Pleasantness begins at the skin, but it is not settled there. Mechanosensory and thermosensory afferents encode stimulus features, and only later, through spinal filtering, interoceptive integration, and appraisal, do those features acquire hedonic tone (Rolls and Grabenhorst, 2008). Aβ pathways form the primary neural substrate of discriminative touch processing, carrying fast information about location, pressure, texture, vibration, and movement across the skin (Abraira and Ginty, 2013; McGlone et al., 2014). It would be a mistake to redescribe them as intrinsically affective. Their role is more indirect, but no less important: they give touch its shape, timing, and tactile grammar, which can then be evaluated in relation to expectation, memory, context, and learned preference. This squares with the observation that glabrous skin, where CT afferents are sparse, can still support pleasant touch under the right conditions (Marshall and McGlone, 2020; Case et al., 2023). The point is not to oppose discriminative and affective touch too neatly. Aβ pathways may not convey pleasantness as such, yet they help determine what is available to become pleasant.

CT afferents occupy a different place in the story. They are slow, unmyelinated fibers found mainly in hairy skin, tuned to gentle stroking in the approximate range of 1–10 cm/s and especially responsive to temperatures close to skin warmth (Löken et al., 2009; Ackerley et al., 2014). CT-responsive pathways project to posterior insula and related interoceptive regions, which helps explain their importance for affective touch (Morrison et al., 2010; Liljencrantz and Olausson, 2014; Davidovic et al., 2019). Yet CT signaling alone does not decide whether touch feels good. Who touches, why, with what expectation, and with what sense of safety can change the experience. A technically CT-optimal stimulus may feel soothing in a trusted context and intrusive in an ambiguous or threatening one (Ellingsen et al., 2015; Saarinen et al., 2021). This is where the model has to stay concrete: the peripheral channel matters, but the person receiving the touch is not a passive recording device.

Temperature adds another layer. Warmth tends to increase reported ease, but not because warmth carries a simple hedonic code. Thermosensory signaling, including TRPV1- and TRPM8-related pathways, interacts with vascular tone, arousal, thermal comfort, interoceptive prediction, and learned associations of care (Caterina and Julius, 2001; Ackerley et al., 2014; Pertusa et al., 2023; Eto et al., 2024; Vabba et al., 2025). CT firing itself is enhanced by skin-like warmth, so thermal and tactile channels are already entangled before the experience is consciously evaluated. The mapping from stimulus intensity to pleasure is also unstable. Deep pressure, cold exposure, and thermal contrast can recruit conditioned pain modulation and relief-related reward when the intensity is bounded, predictable, and under the recipient’s control (Leknes and Tracey, 2008; Field, 2014; Bitar et al., 2018; Sirucek et al., 2021; Karcz et al., 2024). Under those conditions, even nociceptive input can be folded into a positive experience (Leknes et al., 2013). The same cold plunge that feels punishing when imposed may feel bracing, even rewarding, when chosen, timed, and followed by recovery.

The dorsal horn is a primary site of signal convergence. Inhibitory and excitatory interneurons integrate Aβ, CT, thermal, and nociceptive signals, shaping what is transmitted to higher centers and allowing non-nociceptive touch to dampen nociceptive signaling (Abraira and Ginty, 2013; Braz et al., 2014; Chirila et al., 2022). Specialized channels clearly exist, but specialization is only the beginning of the story. Peripheral input becomes hedonic as it is filtered by spinal gating, descending control, bodily state, and the meaning attached to the encounter.

## Spinal and subcortical processing

Touch, heat, and nociception enter circuits that regulate pain transmission, defensive state, and autonomic tone. These circuits are not simply conduits from skin to cortex. They help determine whether stimulation is amplified, inhibited, ignored, transformed into relief, or experienced as aversive. Gate Control Theory remains useful because it explains why rubbing, pressure, or massage can reduce pain by engaging large-diameter non-nociceptive input and inhibitory interneurons in the dorsal horn (Melzack and Wall, 1965). Contemporary circuit work has made this picture more differentiated, identifying inhibitory and excitatory dorsal horn interneuron classes, parallel ascending pathways, and descending inputs that vary with context and state (Abraira and Ginty, 2013; Braz et al., 2014; Chirila et al., 2022; Karcz et al., 2024). The old gate metaphor still works, but the gate has more hinges than the original model could show.

In massage, bathing, sauna, or contrast rituals, spinal gating is part of the story but unlikely to be the whole of it. Local inhibition, descending appraisal, expectation, and current physiology all influence whether a stimulus is soothing or intrusive. Firm pressure, cold exposure, or thermal contrast may become relieving when the person has consented to the experience, understands its limits, and expects a beneficial outcome (Bitar et al., 2018; Sirucek et al., 2021; Karcz et al., 2024). Endogenous opioids contribute to the feeling of relief (Sirucek et al., 2021), while expectation, controllability, and appraisal influence whether that relief becomes pleasurable, merely tolerable, or absent (Amanzio and Benedetti, 1999; Wager et al., 2004; Barrett and Simmons, 2015).

The PAG-RVM network is central to descending pain control and defensive regulation. It can suppress or facilitate nociceptive transmission depending on state, expectation, and perceived threat (Loyd and Murphy, 2009; Tracey and Bushnell, 2009; Zhang et al., 2024). Its neurochemistry is unlikely to be uniform. Relief from bounded discomfort may be relatively opioid-weighted; gentle affiliative contact may draw more strongly on oxytocin and safety signaling; repeated engagement with valued routines may recruit dopaminergic learning (Leknes and Tracey, 2008; Le Merrer et al., 2009; Uvnas-Moberg et al., 2014; Berridge and Kringelbach, 2015; Ellingsen et al., 2015).

Brainstem autonomic nuclei, including the nucleus tractus solitarius, parabrachial nucleus, and reticular formation, integrate somatosensory and interoceptive cues and influence sympathetic-parasympathetic balance (Craig, 2002; Forstenpointner et al., 2022). Under safe conditions, tactile and thermal input may be accompanied by lower heart rate, reduced stress markers, and shifts toward parasympathetic regulation (Diego and Field, 2009; Charkoudian, 2010; Duarte and Pinto-Gouveia, 2017; Huber et al., 2025). We caution against reading too much into HRV or heart rate alone. Similar profiles can reflect reduced threat, attentional drift, felt safety, or active regulation, and the distinction often depends on context and report.

## Cortical and central mechanisms: from sensation to valuation

Once tactile and thermal signals reach cortical and limbic networks, they are no longer processed only as location, intensity, or duration. They are evaluated as events happening to a particular body, in a particular context, with anticipated consequences. Primary and secondary somatosensory cortices encode location, intensity, timing, texture, and movement across the skin (Delhaye et al., 2018). These regions help distinguish a slow stroke from vibration, deep pressure from superficial contact, and diffuse warmth from focal heat. Hedonic value, however, depends on how this sensory detail is combined with interoceptive, limbic, and prefrontal processes (Craig, 2002; Morrison et al., 2010).

The insula is especially important because it links touch and temperature to the felt condition of the body. Posterior insula integrates tactile, thermal, and visceral signals; mid- and anterior insula contribute to awareness and affective appraisal (Craig, 2002; Liljencrantz and Olausson, 2014). CT-related input and warmth may be felt as bodily ease when interoceptive cues fit expectations (Crucianelli and Ehrsson, 2023; Vabba et al., 2025). But the insula is not a hedonic switch. It works with cingulo-opercular, limbic, and prefrontal partners to organize bodily state and meaning.

The anterior cingulate cortex adds salience, effort, and affective-motivational evaluation. Dorsal ACC is more often associated with conflict and aversive salience, whereas pregenual and rostral regions are more often linked to positive affect and safety states (Francis et al., 1999; Rolls et al., 2003). This gradient helps explain why similar input can be read as threat in one setting and care in another. Predictability, controllability, and interpersonal alignment change what the stimulus seems to require (Mazza et al., 2023).

Reward circuitry gives tactile and thermal experience a history. Mesolimbic dopamine is involved in reward prediction, motivation, and the learning of routines that reliably bring relief or comfort (Berridge and Kringelbach, 2015; Schultz, 2015; Lewis et al., 2021). Oxytocinergic signaling, particularly in affiliative contexts, may increase felt safety and trust, thereby amplifying the pleasurable qualities of gentle touch (Morhenn et al., 2008; Baskerville and Douglas, 2010; Uvnas-Moberg et al., 2014). One open question is how far professional touch can leverage these affiliative systems without the broader social context that usually supports oxytocin release.

Cognitive reappraisal and expectancy can be placed within the same loop. Verbal reassurance, professional framing, familiar ritual sequence, and prior positive experience may bias vmPFC/OFC and predictive-processing systems toward a benign interpretation of bodily input; this, in turn, can alter ACC salience, insular precision weighting, PAG-RVM modulation, and subsequent autonomic recovery. In this formulation, expectancy is neither outside the body nor identical with placebo. It is one route through which meaning changes bodily regulation.

One example of a more explicit MIHR pathway can be stated anatomically. Tactile and thermal signals may reach posterior and then anterior insula, where bodily state is represented as interoceptive feeling; ACC may assign salience and regulatory demand; vmPFC/OFC may integrate expected benefit, context, and subjective value; NAc and related mesolimbic structures may support anticipation, reinforcement, and willingness to repeat; and PAG-RVM and hypothalamic-brainstem pathways may translate appraisal into descending pain modulation and autonomic output. This sequence is not meant as a single serial pipeline. Rather, it illustrates how interoceptive valuation, reward learning, descending modulation, and autonomic regulation can form a recurrent loop.

Thermal signals do not form a separate stream. Warmth during massage or bathing may enhance comfort by aligning thermoregulatory, interoceptive, and contextual expectations of safety (Crucianelli and Ehrsson, 2023). Conversely, cold, heat, or firm pressure may first increase salience and then produce relief when the stimulus is predictable, voluntary, and clearly time-limited (Leknes and Tracey, 2008; Field, 2014; Bitar et al., 2018). Pleasantness depends on how sensation is integrated with bodily state, reward history, appraisal, and expectation (Kringelbach and Berridge, 2017). This is why the same intervention can feel medicinal, indulgent, invasive, or pointless, depending on how the body and the situation are read.

## Neurochemical and hormonal modulation

Neurochemical systems influence how tactile and thermal inputs are felt, reinforced, and remembered. Endogenous opioids, dopamine, oxytocin, serotonin, stress hormones, and cellular stress responses each contribute, but none tells the whole story on its own.

Endogenous opioids provide an important bridge between analgesia and relief-related pleasure. β-endorphins and related peptides act within PAG-RVM-spinal networks and limbic structures to inhibit nociceptive transmission and alter affective responses to discomfort (Le Merrer et al., 2009; Loyd and Murphy, 2009; Field, 2014). Heat and cold alter pain-temperature interactions, and the offset of controlled discomfort can itself be rewarding (Morin et al., 1999; Bitar et al., 2018; Chabal, 2021; Zhang et al., 2024). In the present framework, opioids are therefore not simply analgesic. They help explain how the end of discomfort can acquire positive affective value, especially when the episode was chosen, expected, and safely contained.

The precise role of opioids in liking, relief, and pain reduction remains debated (Berridge and Robinson, 2016). A useful working distinction is that opioid systems contribute to hedonic impact and relief, whereas dopamine tracks incentive salience, anticipation, and reinforcement learning. In practice, the boundary is not clean. Safety, predictability, and trust may amplify opioid-related relief, while uncertainty or loss of control may blunt it (Ellingsen et al., 2015; Sirucek et al., 2021).

Dopamine is most relevant to anticipation, motivation, and repeated engagement. Familiar music, scent, lighting, and ritual sequence may shape reward predictions before any physical contact or heat exposure begins (Bromberg-Martin et al., 2010). Alternating thermal exposures may also pair manageable challenge with relief, strengthening reinforcement when recovery is experienced as beneficial (Mitchell et al., 2024). Oxytocin is more closely tied to social meaning and safety. It responds to some forms of gentle affiliative touch and may support trust, bonding, and a care-related interpretation of contact (Insel, 1992; Uvnas-Moberg et al., 2014; Li et al., 2022). Professional touch may recruit parts of this system, especially with good attunement and explicit consent, but may rely more heavily on predictability and ritual cues than intimate touch does (Morhenn et al., 2008; Walker et al., 2022).

Serotonin likely contributes more indirectly, as part of affective stability and stress resilience rather than as the immediate generator of tactile pleasure (Cools et al., 2008). Massage studies reporting increased serotonin and dopamine alongside reduced cortisol are consistent with improved affect and lower stress, but peripheral measures do not map cleanly onto central neurotransmission (Field et al., 2005; Field, 2010). The HPA axis is another part of this regulatory background: supportive touch and thermal practices are often associated with reduced cortisol (Field et al., 2005; Uvnas-Moberg et al., 2014; Dreisoerner et al., 2021). Lower stress may clear the way for comfort by increasing reward sensitivity and facilitating oxytocinergic or opioid processes, but it is not the same as pleasure.

Thermal interventions also engage slower cellular stress responses, including heat-shock protein activity and related adaptive pathways (Hunt et al., 2019; Patrick and Johnson, 2021; Hu et al., 2022). These processes may support perceived recovery over days to weeks and should be kept conceptually separate from the immediate pleasure of warmth or post-cold relief. The neurochemical profile of comfort is therefore likely to vary by practice and context. Opioids, dopamine, oxytocin, serotonin, HPA-axis change, and heat-shock cascades may all contribute, but in different proportions and on different timescales. This, however, is a shorthand, not a strict division of labor.

## Autonomic and cardiovascular modulation: regulation, readout, or driver of pleasure?

Touch and temperature alter autonomic state through brainstem and hypothalamic circuits. These shifts help many people feel calmer, but they do not, by themselves, explain why something feels good (Porges, 2007). Affective touch is often accompanied by increased HRV, lower heart rate, and reduced cardiovascular reactivity, especially when the setting is safe and predictable (Duarte and Pinto-Gouveia, 2017; Forstenpointner et al., 2022; Huber et al., 2025). Safety appraisals and interpersonal attunement make parasympathetic engagement more likely, though not guaranteed.

At the same time, HRV and related markers need careful handling. They reflect respiration, baroreflex function, cardiac regulation, posture, movement, and central autonomic control, not hedonic value itself (Thayer and Lane, 2009; Critchley and Harrison, 2013; Laborde et al., 2017). Studies should therefore report respiratory and postural conditions wherever possible and treat repeated autonomic observations with models that account for subject-level variability. HRV becomes more informative when interpreted alongside what happened, where it happened, and how it felt. Warm exposure recruits thermoregulatory circuitry centered in the preoptic hypothalamus, promotes vasodilation, and reduces sympathetic vasoconstrictor tone; in supportive contexts, these changes are often accompanied by comfort and relaxation (Charkoudian, 2003; Lee et al., 2011; Li et al., 2023). Warmth therefore works on two fronts: it changes vascular state and evokes associations of shelter, care, and temporary withdrawal from demands.

This interpretation is compatible with neurovisceral integration accounts, in which prefrontal and cingulate systems exert flexible inhibitory control over subcortical and brainstem autonomic circuits, and with autonomic-space approaches that distinguish sympathetic activation, ventral-vagal regulation, and defensive shutdown rather than treating arousal as a single dimension (Porges, 2007; Thayer and Lane, 2009). For the MIHR, this means that a soothing response should not be defined simply as low arousal. A person may show relaxed engagement, alert pleasure, effortful tolerance, or withdrawal with superficially similar heart-rate values. Autonomic measures therefore need to be interpreted in relation to phase, subjective report, respiration, posture, and perceived safety.

Cold exposure follows a different temporal pattern. It initially increases sympathetic activation, vasoconstriction, and arousal, but may be followed by vigor, relief, or calm after the stimulus ends (Charkoudian, 2010). Alternating heat and cold exploits this activation-recovery swing and likely taps vascular responsiveness, interoceptive prediction, endogenous modulation, and the reward that attends the offset of discomfort (Mitchell et al., 2024). The popular language of reset is imprecise, but it captures a real phenomenological transition from challenge to recovery.

Massage engages somato-autonomic pathways through low-threshold mechanoreceptors and sustained pressure. Firm pressure, predictably delivered and responsive to cues, often supports relaxation; poorly attuned or excessive pressure can do the opposite (Diego and Field, 2009; Field, 2014). The same physical intensity can therefore produce different autonomic consequences depending on how it is delivered and interpreted. Limbic and paralimbic regions help coordinate this response. Insula-amygdala-hypothalamus-brainstem loops organize bodily state, while the ACC contributes to salience, regulation, and readiness for action (Crucianelli and Ehrsson, 2023; Mazza et al., 2023). The influence is bidirectional: appraisal shapes autonomic output, and interoceptive feedback changes what the stimulus feels like.

Warmth, low lighting, quiet, predictable sequence, and therapeutic alliance can all support safety appraisals and parasympathetic engagement (Saarinen et al., 2021; Cheong et al., 2023). These are not mere trappings; they help shape what the body takes to be happening. Autonomic shifts belong inside the explanation, but not at its center. They contribute to calm and may stabilize restorative states; they cannot, by themselves, account for why stimulation becomes pleasant.

## Psychosocial, cultural, developmental, and evolutionary modulators: meaning as a biological variable

Touch is relational even in clinics and spas; heat and water arrive in cultural containers. Meaning is not an afterthought. It shifts prediction, attention, descending control, neurochemistry, and autonomic tone (Clark, 2013; Seth, 2013). Empirical studies show that expectation, attention, and emotional state influence both subjective experience and neural responses to tactile and nociceptive stimuli (Croy et al., 2019). These effects vary across individuals, depending on prior experience, sensory sensitivity, and cognitive-affective traits (Saarinen et al., 2021; Li et al., 2022).

Developmentally, touch regulates physiological and emotional state from the beginning of life. Skin-to-skin contact stabilizes arousal and supports attachment and affect regulation (Field, 2010; Cascio et al., 2019). Early tactile experience may tune insular-limbic circuitry involved in interoception and stress buffering, biasing some individuals to find slow, gentle touch comforting later in life (Gordon et al., 2013; McGlone et al., 2014; Croy et al., 2019). At the same time, attachment history, sensory sensitivity, neurodevelopmental variation, and context introduce real heterogeneity. Early touch shapes tendencies, not destinies (Saarinen et al., 2021; Fotopoulou et al., 2022). This qualification matters clinically: the same slow touch that carries safety for one body may carry intrusion for another.

Kangaroo mother care provides a well-documented example of tactile regulation in a broader multisensory setting. It combines skin-to-skin contact with warmth, rhythmic stimulation, smell, heartbeat, vocal cues, and relational co-regulation (Bieleninik et al., 2021; Kostilainen et al., 2021). Evolutionary accounts similarly emphasize grooming as a means of social cohesion, stress reduction, and alliance maintenance, with oxytocinergic and dopaminergic systems contributing to affiliative reward (Dunbar, 2010; Uvnas-Moberg et al., 2014; Griesser et al., 2025). Professional touch is not grooming by another name. It may exchange intimacy for explicit consent, predictability, ritual structure, and skilled attunement: another route to safety, but not the same one.

Culture gives these bodily practices recognizable forms. Finnish sauna, Japanese onsen, Turkish hammam, and Russian banya combine heat, water, sequence, social convention, and narratives of purification, recovery, endurance, or belonging (Tsonis, 2016; Laukkanen et al., 2018; Poluzzi and Esposito, 2019). These forms teach participants how to anticipate and interpret sensations. This does not mean that culture creates the physiology from nothing. Rather, culturally patterned practices arrange bodily inputs, including heat, touch, smell, quiet, rhythm, covering or nudity, and social presence, so that thermosensory and interoceptive signals become legible as cleansing, restoration, conviviality, discipline, or care (Moerman and Jonas, 2002; Price et al., 2008; Matsumoto and Hwang, 2012; Barrett, 2017). The physiology is real, but it comes already dressed in a practice.

The language of cleanliness, purification, and bodily reset deserves particular attention. In bathing and washing rituals, symbolic meaning and interoception are difficult to separate. Disgust and contamination-avoidance systems, involving regions such as the insula and ACC, may shift from aversive vigilance toward relief and safety when cues of cleansing, warmth, and social acceptance are present (Calder et al., 2001; Wright et al., 2004; Oaten et al., 2009; Curtis et al., 2011). The pleasure of feeling clean may therefore resemble relief-related pleasure in pain modulation: positive affect rides on the resolution of an aversive state (Rozin et al., 2008).

Placebo and expectancy research offers one of the clearest demonstrations that meaning has biological effects. Expectation-driven analgesia can involve endogenous opioid release and altered brain activity during anticipation and experience (Amanzio and Benedetti, 1999; Wager et al., 2004; Benedetti, 2006; Price et al., 2008). Touch and thermal therapies live inside cue-rich environments: professional framing, ritual order, interpersonal reassurance, anticipated benefit, and recognizable sensory sequences. Expectancy is therefore not a nuisance to be removed from the explanation, but one pathway by which these practices work.

The distinction between hedonia and eudaimonia is useful here. Hedonia refers to felt pleasure, relief, and comfort in the moment, while eudaimonia refers to broader wellbeing linked to meaning, agency, connectedness, and valued functioning (Reybrouck and Eerola, 2022). A massage or sauna session may be hedonically pleasant because it feels warm, safe, and relieving. Repeated practice may also become eudaimonic when it supports recovery routines, self-care, social connection, or a sense of inhabiting the body more securely. The two forms of wellbeing can reinforce one another, but they should not be collapsed.

Interpersonal processes sharpen this point. Therapeutic alliance, pacing, and biobehavioral synchrony, such as coordinated breathing or matched rhythm, may enhance oxytocinergic and parasympathetic engagement (Prause et al., 2021; McParlin et al., 2022). Conversely, unwanted, ambiguous, or poorly attuned touch can evoke defensive responses. In supportive settings, inward attention can make warmth, pressure, or relief more salient. In chronic pain, anxiety, trauma, or heightened sensory sensitivity, the same inward focus may intensify discomfort (Fusaro et al., 2022; Ackerley, 2023; Crucianelli and Ehrsson, 2023; Haenen et al., 2024). For that reason, attention to sensation should be tailored rather than assumed to help everyone. Meaning has biological effects because it alters appraisal, autonomic regulation, descending modulation, and neurochemical tone.

## Integration of mechanisms and hedonic experience: a multilevel integrative model of hedonic regulation

**Figure 1:**
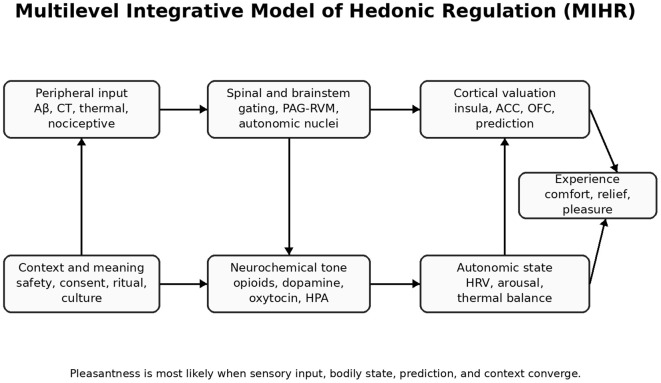
Schematic representation of the multilevel integrative model of hedonic regulation (MIHR). The model depicts tactile and thermal pleasure as an emergent outcome of interactions among peripheral signaling, spinal and brainstem modulation, cortical evaluation, neurochemical reinforcement, autonomic state, and psychosocial meaning.

The MIHR brings these processes into one explanatory frame (**Figure 1**). Its premise is modest but useful: pleasure, analgesia, and bodily settling may arise from interactions among skin and spinal processing, cortical evaluation, neurochemistry, autonomic shifts, and the social and cultural frames that make the experience legible (Leknes and Tracey, 2008; Barrett and Simmons, 2015; Berridge and Kringelbach, 2015; Ellingsen et al., 2015). The model does not replace specific accounts of CT afferents, gate control, opioid analgesia, thermoregulatory physiology, or expectancy. It asks how they fit together in lived practice, where pressure has a rhythm, warmth has a setting, and relief has a meaning. Operationally, each study should specify the input being manipulated, the phase being measured, the pathway being tested, and the outcome of interest.

To keep the model from becoming too diffuse, the MIHR distinguishes four roles. Tactile and thermal parameters are proximal inputs. Spinal gating, descending modulation, cortical appraisal, autonomic state, and neurochemical change are candidate pathways through which experience is shaped. Safety, expectancy, consent, attachment history, trauma exposure, sensory sensitivity, cultural meaning, and therapist attunement may alter the strength or direction of these pathways. Pleasantness, relief, calm, pain reduction, recovery meaning, and willingness to repeat the practice are outcomes. These roles are not fixed once and for all, but empirical tests should specify them in advance. **Box 2** illustrates how broad constructs such as safety, anxiety, bodily confidence, or relief may occupy different causal positions depending on the hypothesis, phase of treatment, and level of analysis. The same construct can appear in different places only if the design justifies it. For example, perceived safety may be measured before the intervention as a moderator, repeatedly during stimulation as a mediator, or after recovery as an outcome. **Box 2** should therefore be read as a template for pre-specification rather than as a claim that every study should estimate every path.

Box 2 **Context-dependent causal roles of MIHR constructs.**

**Table:** 

Model element	Example operationalization	Illustrative path-analysis role
Input	Pressure, stroking velocity, skin site, warmth/cold, duration	Exogenous manipulated variables
Early pathway	Aβ/CT signaling, thermal signaling, dorsal-horn gating, PAG-RVM modulation	Candidate mediators of sensory and analgesic effects
Central valuation	Anterior insula, ACC, vmPFC/OFC, NAc, expectancy or reappraisal	Candidate mediators linking bodily input to value and prediction
Autonomic pathway	HRV, heart rate, blood pressure, skin conductance, respiratory control	Candidate mediator or physiological readout, depending on timing
Context/person variables	Consent, perceived control, trust, cultural familiarity, trauma history, sensory sensitivity	Moderators or mediated moderators
Outcomes	Pleasantness, relief, pain reduction, calm, recovery meaning, willingness to repeat	Primary or secondary dependent variables

**Box 3** then gives concrete examples of how these roles can be translated into causal hypotheses without turning the MIHR into a rigid statistical template.

Box 3 **Examples of causal roles within the MIHR**

**Table:** 

Example	Interpretation
Mediation	Firm pressure or CT-optimal stroking may increase pleasantness partly through spinal gating, interoceptive appraisal, endogenous modulation, or parasympathetic engagement.
Moderation	The same stimulus is expected to feel better when trust, consent, predictability, and control are high, and worse when ambiguity or threat dominates.
Moderated mediation	Massage may increase pleasantness through perceived safety and autonomic settling, but this pathway may be weaker among people with trauma exposure, chronic pain, high sensory sensitivity, or insecure attachment.
Mediated moderation	Attachment history or therapist attunement may alter hedonic response partly by changing momentary trust and felt safety.
Bidirectional coupling	Pleasantness may follow autonomic settling, but pleasant appraisal may also feed back to reduce arousal; repeated measurement is needed to separate sequence from correlation.

Safety is the clearest example of this role-dependence. Before stimulation, trait-like safety expectations, trauma history, or trust in the therapist may moderate the effect of identical pressure or temperature. During stimulation, momentary felt safety may mediate the pathway from predictable, consented contact to autonomic settling and reduced threat appraisal. After stimulation, safety may be part of the outcome, expressed as calm, bodily confidence, or readiness to repeat. The model is therefore multilevel, but each empirical test should assign safety, anxiety, depression, anger, or boredom to a specific causal role for that test.

At the skin, Aβ and CT fibers encode different features of touch; thermoreceptors register warmth and coolness; nociceptors respond to high-intensity stimulation (Abraira and Ginty, 2013; McGlone et al., 2014). In the dorsal horn, interneuronal networks integrate these signals and can suppress nociceptive transmission when non-noxious touch is present (Melzack and Wall, 1965; Braz et al., 2014; Chirila et al., 2022). This provides one biological route by which reduced pain and positive bodily sensation often appear together. Analgesia, however, does not guarantee pleasure. Relief becomes rewarding when it is noticeable, expected, and meaningful to the person receiving it.

Ascending signals engage the insula, ACC, orbitofrontal cortex, thalamus, and limbic networks, where bodily signals are evaluated in relation to comfort, safety, threat, and expected outcome (Craig, 2002; Rolls et al., 2003; Crucianelli and Ehrsson, 2023). Predictive processing is useful here, as long as it is not asked to explain everything. The brain maintains expectations about bodily state, and incoming signals update or confirm those expectations (Barrett and Simmons, 2015; Kleckner et al., 2017). Gentle touch and warmth may reduce uncertainty and allow parasympathetic engagement. Still, reinforcement learning, interpersonal meaning, and cultural scripts influence which predictions matter and what counts as a good outcome (Matsumoto and Hwang, 2012).

Endogenous opioid, dopaminergic, and oxytocinergic systems may contribute differentially to relief, anticipation, reinforcement, and affiliative safety, although they interact and should not be mapped one-to-one onto subjective states (Le Merrer et al., 2009; Uvnas-Moberg et al., 2014; Berridge and Kringelbach, 2015). Autonomic embodiment gives the process bodily weight. Parasympathetic shifts, including increases in HRV and reductions in cardiovascular arousal, often accompany calm and safety when the context supports non-threat (Gordon et al., 2013; Duarte and Pinto-Gouveia, 2017; Forstenpointner et al., 2022; Huber et al., 2025). Through interoceptive feedback, these bodily changes loop back to evaluation systems and shape how touch and temperature are felt (Thayer and Lane, 2009; Critchley and Harrison, 2013). In our view, context is not ornamentation. It is one of the ways stimulation becomes safe, tolerable, or pleasant.

## Testable predictions

With this causal vocabulary in place, the MIHR suggests a set of testable ideas, best approached with cautious manipulations rather than one-size-fits-all protocols. Prediction 1: when pressure, stroking velocity, temperature, and duration are held constant, positive therapeutic framing and higher perceived safety should increase pleasantness and relief ratings relative to neutral or ambiguous framing, at least for some participants (Ellingsen et al., 2015). Prediction 2: the balance between CT- and Aβ-mediated contributions should depend on skin type and framing. On hairy skin, CT-optimal stroking should be especially pleasant when social meaning is supportive; on glabrous skin, pleasantness may track discriminative features and learned associations more closely (Löken et al., 2009; Ellingsen et al., 2015; Case et al., 2023).

Prediction 3: relief-related pleasure should peak when mild discomfort is predictable, controllable, and clearly time-limited; subjective relief should covary with markers of endogenous modulation and physiological recovery, especially under conditions that preserve agency and safety (Leknes and Tracey, 2008; Bitar et al., 2018; Sirucek et al., 2021). Prediction 4: HRV, skin conductance, and blood pressure will probably predict pleasantness only imperfectly. They become more informative when paired with ratings of safety, expectation, bodily awareness, and perceived control (Craig, 2002; Thayer and Lane, 2009; Critchley and Harrison, 2013; Ellingsen et al., 2015; Laborde et al., 2017). Prediction 5: individual differences should help organize response profiles: attachment history, chronic pain, anxiety, trauma exposure, neurodevelopmental variation, and interoceptive sensitivity may identify subgroups for whom the same intervention is soothing, neutral, or aversive (Cascio et al., 2019; Saarinen et al., 2021; Fusaro et al., 2022; Li et al., 2022; Haenen et al., 2024).

Finally, cue-only or expectation-only conditions should be included more often. Such arms can estimate how much of the response is generated before or around the stimulus rather than by the sensory input alone (Amanzio and Benedetti, 1999; Wager et al., 2004; Benedetti, 2006; Price et al., 2008). The point is not to turn the model into a statistical exercise. It is to make the assumptions explicit enough that narrower accounts can be fairly tested against it. Pre-specified causal diagrams, factorial manipulations, longitudinal timing, mixed-effects models for repeated physiological observations, and responder profiles are useful tools for that purpose (Pearl, 2009; Hayes, 2018; Hernán and Robins, 2020; Kline, 2023).

One practical way to test the MIHR is to compare it directly with narrower explanations of affective touch and thermal pleasure. **Table 1** summarizes how several such models differ in their core predictions and what findings would challenge them.

**Table 1:** How the MIHR can be tested against narrower models.

Model	Core prediction	What would challenge the model
CT-dominance model	Pleasantness is mainly predicted by CT-optimal stimulation, especially on hairy skin.	Strong effects of trust, framing, or perceived safety under identical stimulation would show that CT signaling is insufficient on its own.
Autonomic-regulation model	Higher HRV, lower arousal, or faster recovery should predict higher pleasantness.	Similar autonomic profiles with divergent pleasure ratings would challenge autonomic sufficiency.
Placebo/expectancy model	Framing, anticipated benefit, and cue-only effects should account for much of comfort or relief.	Persistent effects after expectancy is controlled, or effects that vary by skin type, pressure, temperature, or nociceptive offset, would show that sensory and bodily mechanisms remain necessary.
Reward/neurochemical model	Opioid-, dopamine-, and oxytocin-related processes should explain relief, liking, and repeated engagement.	Divergent hedonic responses despite similar analgesic or reward markers would require attention to context, appraisal, and personal history.
MIHR	Pleasantness is best predicted by the joint pattern of sensory input, bodily state, contextual meaning, and individual history.	MIHR would be weakened if contextual and person-level variables do not improve prediction beyond simpler single-mechanism models. A stronger disconfirmation would be repeated failure of prespecified context/person-level terms to add cross-validated predictive value or theoretically coherent interaction effects beyond narrower models.

The limits of the model should also be stated plainly. The MIHR is a heuristic integrative framework, not yet a validated mechanistic system. Not all pathways will be active in all practices or in all people, and some constructs, such as felt safety, cultural familiarity, or bodily confidence, are harder to measure than pressure, temperature, or HRV. Because the model is broad, it could overfit findings after the fact unless hypotheses are specified in advance. Its value will depend on whether it helps researchers make better predictions, not on whether it can accommodate every result after the event.

For falsifiability, the MIHR requires study-specific quantitative benchmarks rather than a universal cut-off. In adequately powered confirmatory studies, one conservative benchmark would be that contextual and person-level variables should improve out-of-sample prediction by a prespecified amount, for example by reducing prediction error by at least 5%, improving information criteria relative to a single-mechanism model, or adding approximately 2-3% explained variance for subjective outcomes beyond sensory, CT, autonomic, or expectancy-only predictors. For interaction or moderated-mediation tests, smaller increments may still be meaningful, but they should be declared in advance, for example ΔR^2^ of about 1% or a standardized indirect or interaction effect of approximately 0.10-0.20 when theory and measurement reliability justify that threshold. Conversely, the MIHR would be weakened if preregistered multilevel terms repeatedly fail to improve cross-validated prediction, show unstable signs across samples, or add only *post hoc* explanatory complexity without better prediction of pleasantness, relief, or willingness to repeat.

## Future directions and conclusion: toward a multilevel science of therapeutic touch

Future studies should trace interactions among levels without letting methodology displace the phenomenon itself. Multimodal studies combining fMRI or PET with autonomic measures, endocrine markers, quantitative sensory testing, and interoceptive self-report would better capture how sensory integration, evaluation, neurochemistry, and autonomic embodiment unfold together (Becker et al., 2019; Sirucek et al., 2021). Where feasible, designs should separate baseline, cue or anticipation, stimulation, offset, and recovery, because the same marker can mean different things at different moments. Standardized and preregistered protocols should help address the heterogeneity seen in meta-analyses of touch interventions (Packheiser et al., 2024).

Individual variability should be treated as signal rather than noise. Latent-profile or responder-phenotype approaches could identify groups such as safety-context responders, relief-dominant responders, or individuals who rely more on discriminative tactile structure. Such profiles may eventually guide more tailored matching of techniques and settings (Cascio et al., 2019; Croy et al., 2019; Poluzzi and Esposito, 2019; Li et al., 2022). Researchers should also attend to selection bias, expectancy bias, cultural bias, dropout or adherence effects, and therapist effects, since each may shape both the sensory intervention and the meaning attached to it (Podsakoff et al., 2003).

Ecological validity is equally important. Touch and heat are rarely delivered in isolation. Massage comes with warmth, scent, quiet, pacing, and expectations; bathing includes sequence, setting, and cultural meaning. Research should therefore test components as well as combinations and sequences, with expectancy and perceived control measured rather than left implicit (Ellingsen et al., 2015; Cheong et al., 2023).

Developmental and translational questions remain open. Longitudinal studies are needed to examine whether repeated engagement with touch or thermal practices changes autonomic flexibility, pain sensitivity, sleep, mood regulation, immune markers, or resilience. Clinical groups, including chronic pain, anxiety, trauma histories, neurodevelopmental differences, and altered somatosensory processing, will likely need adapted protocols rather than standard dosing (Jakubiak and Feeney, 2017; Laukkanen et al., 2018; Haenen et al., 2024).

Future versions of the model should also make room for slower body systems that are only briefly considered here. Endocrine, immune, digestive, and reproductive systems may influence how touch and temperature are experienced through inflammation, metabolic state, hormonal cycling, gut-brain signaling, fatigue, or recovery demands. These systems are unlikely to explain immediate hedonic pleasure on their own, but they may shape baseline bodily state, responder profiles, and longer-term eudaimonic outcomes.

The MIHR proposes that tactile and thermal pleasure emerges from coordinated sensory, regulatory, and contextual processes rather than from sensory input alone. It challenges single-mechanism explanations and brings meaning, context, and bodily prediction into the mechanism rather than leaving them outside (Seth and Friston, 2016). In doing so, it aligns with contemporary models of interoception and predictive regulation, in which perception and bodily state are co-constructed rather than sequentially linked (Barrett and Simmons, 2015; Kleckner et al., 2017). The central claim is therefore not that every level is always active, but that pleasure and relief are most intelligible when the body, the stimulus, and the situation are studied together.

Clinically, this view argues for careful matching between sensory parameters and the person’s context, preferences, and sense of control. Assessing sensory comfort, interoceptive sensitivity, safety cues, and prior touch experiences can guide selection and pacing. Training should emphasize attunement, consent, responsiveness, and thoughtful management of temperature, lighting, sound, sequence, and timing.

More broadly, the enduring appeal of touch- and heat-based practices may reflect their capacity to reduce uncertainty, signal safety, and couple analgesia with reward. This pairing is biologically efficient and experientially compelling: the body is not just stimulated, but reassured, reorganized, and, at times, relieved. Acknowledging the biological role of meaning does not collapse these effects into mere placebo. It permits a more accurate account of how touch and temperature become restorative.

## Data Availability

The original contributions presented in the study are included in the article/supplementary material. Further inquiries can be directed to the corresponding author.
